# Unreliable Tracking Ability of the Third-Generation FloTrac/Vigileo™ System for Changes in Stroke Volume after Fluid Administration in Patients with High Systemic Vascular Resistance during Laparoscopic Surgery

**DOI:** 10.1371/journal.pone.0142125

**Published:** 2015-11-03

**Authors:** Ji-Hyun Chin, Wook-Jong Kim, Jeong-Hyun Choi, Yun A. Han, Seon-Ok Kim, Woo-Jong Choi

**Affiliations:** 1 Department of Anesthesiology and Pain Medicine, Asan Medical Center, University of Ulsan College of Medicine, Seoul, Korea; 2 Department of Anesthesiology and Pain Medicine, School of Medicine, Kyung Hee University, Seoul, Korea; 3 Department of Clinical Epidemiology and Biostatistics, Asan Medical Center, University of Ulsan College of Medicine, Seoul, Korea; University of Florida, UNITED STATES

## Abstract

**Background:**

The FloTrac/Vigileo^™^ system does not thoroughly reflect variable arterial tones, due to a lack of external calibration. The ability of this system to measure stroke volume and track its changes after fluid administration has not been fully evaluated in patients with the high systemic vascular resistance that can develop during laparoscopic surgery.

**Methods:**

In 42 patients undergoing laparoscopic prostatectomy, the stroke volume derived by the third-generation FloTrac/Vigileo^™^ system (SV-Vigileo), the stroke volume measured using transesophageal echocardiography (SV-TEE) as a reference method, and total systemic vascular resistance were evaluated before and after 500 ml fluid administration during pneumoperitoneum combined with the Trendelenburg position.

**Results:**

Total systemic vascular resistance was 2159.4 ± 523.5 dyn·s/cm^5^ before fluid administration. The SV-Vigileo was significantly higher than the SV-TEE both before (68.8 ± 15.9 vs. 57.0 ± 11.0 ml, *P* < 0.001) and after (73.0 ± 14.8 vs. 64.9 ± 12.2 ml, *P* = 0.003) fluid administration. During pneumoperitoneum combined with the Trendelenburg position, Bland-Altman analysis for repeated measures showed a 53.8% of percentage error between the SV-Vigileo and the SV-TEE. Four-quadrant plot (69.2% of a concordance rate) and polar plot analysis (20.6° of a mean polar angle, 16.4° of the SD of a polar angle, and ±51.5° of a radial sector containing 95% of the data points) did not indicate a good trending ability of the FloTrac/Vigileo^™^ system.

**Conclusions:**

The third-generation FloTrac/Vigileo^™^ system may not be useful in patients undergoing laparoscopic surgery, based on unreliable performance in measuring the stroke volume and in tracking changes in the stroke volume after fluid administration during pneumoperitoneum combined with the Trendelenburg position.

## Introduction

Laparoscopic surgery has become increasingly prevalent in diverse major surgical procedures. Moreover, the patients indicated for laparoscopic surgery have become older and have more comorbidities, such as cardiovascular disease with a high illness severity in some patients, than before [1]. Therefore, it might be a substantial issue to monitor the cardiovascular function in those patients in order to achieve appropriate management and finally favorable outcomes. The FloTrac/Vigileo^™^ system (Edwards Lifesciences, Irvine, CA, USA) could be selected for such a purpose, given its accessibility and safety. However, the performance of this system to measure the stroke volume (SV) and track changes in the SV during laparoscopic surgery has not been identified.

The arterial tone should be considered for the FloTrac/Vigileo^™^ system to be adequately used in a specific condition that can alter the arterial tone, because this system does not conduct external calibration [2]. The FloTrac/Vigileo^™^ system has been tried to show a reliable performance for variable arterial tones. Although the third-generation FloTrac/Vigileo^™^ system was developed using a large data set, including septic and liver cirrhosis patients, it still shows an unacceptable performance in the subset of patients who usually showed low systemic vascular resistance (SVR) [3–6]. In contrast, the performance of FloTrac/Vigileo^™^ system has rarely been examined in a high SVR state, such as during laparoscopic surgery.

Therefore, we aimed to investigate the performance of the third-generation FloTrac/Vigileo^™^ system (3.02), including its ability to measure the SV and track changes in the SV after fluid administration, in patients undergoing laparoscopic prostatectomy. We also evaluated the effects of arterial load (both SVR and arterial compliance) on the performance of this system in these patients.

## Materials and Methods

### Patients

The present study was approved by the Asan Medical Center Institutional Review Board (Code 2014–0741), and the requirement for written informed consent was waived because the data were analyzed anonymously. We had previously studied both SV variation and pulse pressure variation during laparoscopic surgery [7]. This study further analyzed the data of patients in our previous study. We initially enrolled 45 patients scheduled for robot (da Vinci^™^ surgical system; Intuitive Surgical, Mountain View, CA, USA)-assisted laparoscopic radical prostatectomy, and finally evaluated 42 patients. Patients with a body mass index > 40 or <15 kg/m^2^, valvular heart disease, arrhythmias, coronary artery disease, a left ventricular ejection fraction < 50%, pulmonary disease, or esophageal disease were excluded.

As described in detail previously [7], an induction of anesthesia was performed using a 4–5 mg/kg of thiopental sodium followed by 0.1 mg/kg of vecuronium. The mechanical ventilation was set to a tidal volume of 8 ml/kg of body weight, a respiratory rate of 8–12 breaths/min to maintain end-tidal CO_2_ between 28 and 35 mmHg, and an inspiratory-to-expiratory time ratio of 1:2. Positive end-expiratory pressure was not used. A 2 *μg*/kg dose of fentanyl was injected before the skin incision. Maintenance of anesthesia was performed with a 1–2 minimum alveolar concentration of sevoflurane with 50% oxygen using medical air.

### Hemodynamic monitoring

A 20-gauge catheter inserted into the radial artery was connected to the transducer of third-generation FloTrac/Vigileo^™^ system, which continuously updates SV every 20 seconds. Before measurement using the third-generation FloTrac/Vigileo^™^ system, the arterial waveform was confirmed not to be dampened by a square wave test, and if this was not optimal, the arterial line and catheter tip were checked to improve the waveform.

### Echocardiographic measurements

One experienced cardiac anesthesiologist performed all the transesophageal echocardiographic examinations using an iE33 Ultrasound System (Philips Ultrasound, Bothell WA, USA)

As described in the previous study [7], the left ventricular outflow tract diameter was measured in a mid-esophageal aortic long-axis view. Aortic blood flow velocities using pulsed wave Doppler were measured in a deep-transgastric long axis view at the same site where the left ventricular outflow tract diameter was measured. The averaged aortic blood flow time velocity integrals of three consecutive respiratory cycles were used to calculate the SV, as follows:

SV = left ventricular outflow tract area * aortic flow time velocity integral

where,

$$\text{left ventricular outflow tract area }=\text{ π*}{\left(\text{left ventricular outflow tract diameter}\right)}^{2}/4$$

### Study protocol

When hemodynamic conditions were stable, measurements were performed at the following 3 points: after anesthetic induction (T_0_), 3 minutes after the steep Trendelenburg position (35°) was added to pneumoperitoneum (insufflation pressure of 15 mmHg) (T_1_), and 3 minutes after infusion of 500 ml colloid (Voluven^®^; Fresenius Kabi, Germany) over 10 minutes using an infusion pump in T_1_ (T_2_).

Each investigator was blinded to the other’s measurement of the SV. At predetermined time points, one investigator measured the echocardiographic variables and another simultaneously measured the following variables; the stroke volume derived by the third-generation FloTrac/Vigileo^™^ system (SV-Vigileo), systolic arterial blood pressures, diastolic arterial blood pressure, mean arterial blood pressure (MABP), and heart rate. Total SVR (TSVR) and arterial compliance were calculated from the measured variables as follows; TSVR was defined as the MABP divided by the SV-TEE and multiplied by 80 [5,8], arterial compliance as the SV-TEE divided by the arterial pulse pressure [8].

During the study protocol, the ventilator settings and anesthetic depth did not change. The study protocol was discontinued when inotropes or vasopressors were required to improve unstable hemodynamics (MABP < 60 mmHg), or an arrhythmia occurred.

### Statistical analysis

Continuous data are expressed as mean ± SD. To evaluate the effect of pneumoperitoneum and the Trendelenburg position on hemodynamic variables, one-way repeated measures analysis of variance (ANOVA) was used. If the one-way repeated measures ANOVA revealed a significant interaction, *post hoc* analysis was performed using Holm-Sidak test. To compare the SV-Vigileo with the SV-TEE at each time point, a Student *t*-test or Mann-Whitney U test was used.

One paired data per patient was collected at T_0_, T_1_, and T_2_. During pneumoperitoneum combined with the Trendelenburg position (both T_1_ and T_2_), Bland-Altman analysis for repeated measures was used to assess the bias (mean difference) and precision (SD of the bias) between the SV-TEE and the SV-Vigileo. In addition, Bland-Altman analysis was used to assess the bias and precision between the SV-TEE and the SV-Vigileo at each time point (T_0_, T_1_, and T_2_, respectively). The percentage error (2SD of the difference divided by the mean SV of the reference method) between the SV-TEE and the SV-Vigileo was assessed, and the tested method was considered interchangeable with the reference method when the percentage error was < 30% [9].

Four-quadrant plot analysis and polar plot analysis were used to examine the trending ability between T_1_ and T_2_ of the third-generation FloTrac/Vigileo^™^ system to accurately track SV changes after fluid administration during pneumoperitoneum combined with the steep Trendelenburg position [10,11]. In four-quadrant plot, we used an exclusion zone of 15% [10,11]. The concordance rate, i.e. the percentage of the number of data points in two of the four quadrants of agreement (right upper and left lower), was considered good when it was above 92% in four-quadrant plot analysis with an exclusion zone of 15% [11]. In polar plot analysis, we used an exclusion zone of 10%, because the mean value of the changes in the SV rather than the length of the hypotenuse of a right-angled triangle formed by the changes in SV was chosen as the magnitude of the changes in the SV [10]; 26 among 42 paired data were analyzed. In polar plot analysis, the acceptance limits of trending ability were (1) a mean polar angle of less than ± 5°, (2) a SD for the polar angle of less than ± 15°, and (3) a radial sector containing 95% of the data points of less than ± 30° [10]. The angular concordance rate, which was the percentage of points within ± 30° radial sector, was considered as good when it was above 95% in polar plot analysis [10].

To evaluate the effect of TSVR or arterial compliance on the difference between the SV-TEE and the SV-Vigileo, the correlation coefficient (CC) was calculated using a Pearson’s correlation test.

A *P* value < 0.05 was considered statistically significant. All statistical analyses were performed using SigmaPlot software, version 12.3 (Systat Software Inc, San Jose, CA, USA), MedCalc version 14.12 (MedCalc Software bvba, Ostend, Belgium), and R statistical software, version 3.1.1. (www.r-project.org).

## Results

Of the 45 patients, 3 were excluded due to the occurrence of arrhythmia or a change to open prostatectomy during the study protocol, and thus 42 patients completed the study protocol. The patient’s demographics and preoperative characteristics are shown in Table 1.

**Table 1:** Demographics and preoperative characteristics.

Age (years)	62.8 ± 7.1 (range, 49–63)
Body mass index (kg/m^2^)	25.2 ± 2.7 (range, 19.7–25.5)
Comorbidities	
Diabetes mellitus	4 (9.5%)
Hypertension	17 (40.5%)
Congestive heart failure	0 (0%)
Peripheral vascular disease	0 (0%)
Mean blood pressure (mmHg)	88.4 ± 9.7
Hematocrit (%)	43.7 ± 2.9
Serum creatinine (mg/dl)	0.9 ± 0.2
Left ventricular ejection fraction (%)	61.7 ± 4.0

Data are presented as mean ± SD or number (percentage).

The hemodynamic variables, including SV measurements and arterial load, are shown in Table 2. The SV-Vigileo were significantly higher than the SV-TEE at all 3 time points (P = 0.022, P < 0.001, and P = 0.003 at T_0_, T_1_, and T_2_, respectively). As regards the arterial load, the TSVR at T_1_ significantly increased compared with T_0_ (P < 0.05), and the TSVR at T_2_ decreased compared with T_1_ (P < 0.05). In contrast, arterial compliance at T_1_ significantly decreased compared with T_0_ (P <0.05).

**Table 2:** Hemodynamic data at different time points.

	T_0_	T_1_	T_2_
SV-Vigileo (ml)	66.5 ± 13.7	68.8 ± 15.9	73.0 ± 14.8* ^†^
SV-TEE (ml)	61.2 ± 15.6	57.0 ± 11.0	64.9 ± 12.2* ^†^
MABP (mmHg)	75.8 ± 9.7	87.5 ± 11.2*	86.6 ± 10.9*
HR (beats/min)	67.4 ± 15.1	60.1 ± 11.7*	62.9 ± 11.6
TSVR (dyn·s/cm^5^)	1604.5 ± 482.0	2159.4 ± 523.5*	1801.9 ± 475.5^†^
AC (ml/mmHg)	1.5 ± 0.4	1.4 ± 0.4*	1.6 ± 0.4^†^

Data are presented as mean ± SD. T_0_, after anesthetic induction in supine position; T_1_, 3 minutes after the steep Trendelenburg position (35°) was added to pneumoperitoneum during which time insufflation pressure was set to 15 mmHg; T_2_, 3 minutes after 500 ml of colloid infusion in T_1_; SV-Vigileo, stroke volume derived by the FloTrac/Vigileo^™^ system; SV-TEE, stroke volume measured using transesophageal echocardiography; MABP, mean arterial blood pressure; HR, heart rate; TSVR, total systemic vascular resistance; AC, arterial compliance.

*P < 0.05 vs T_0_;

^†^ P < 0.05 vs T_1_.

**Fig 1:**
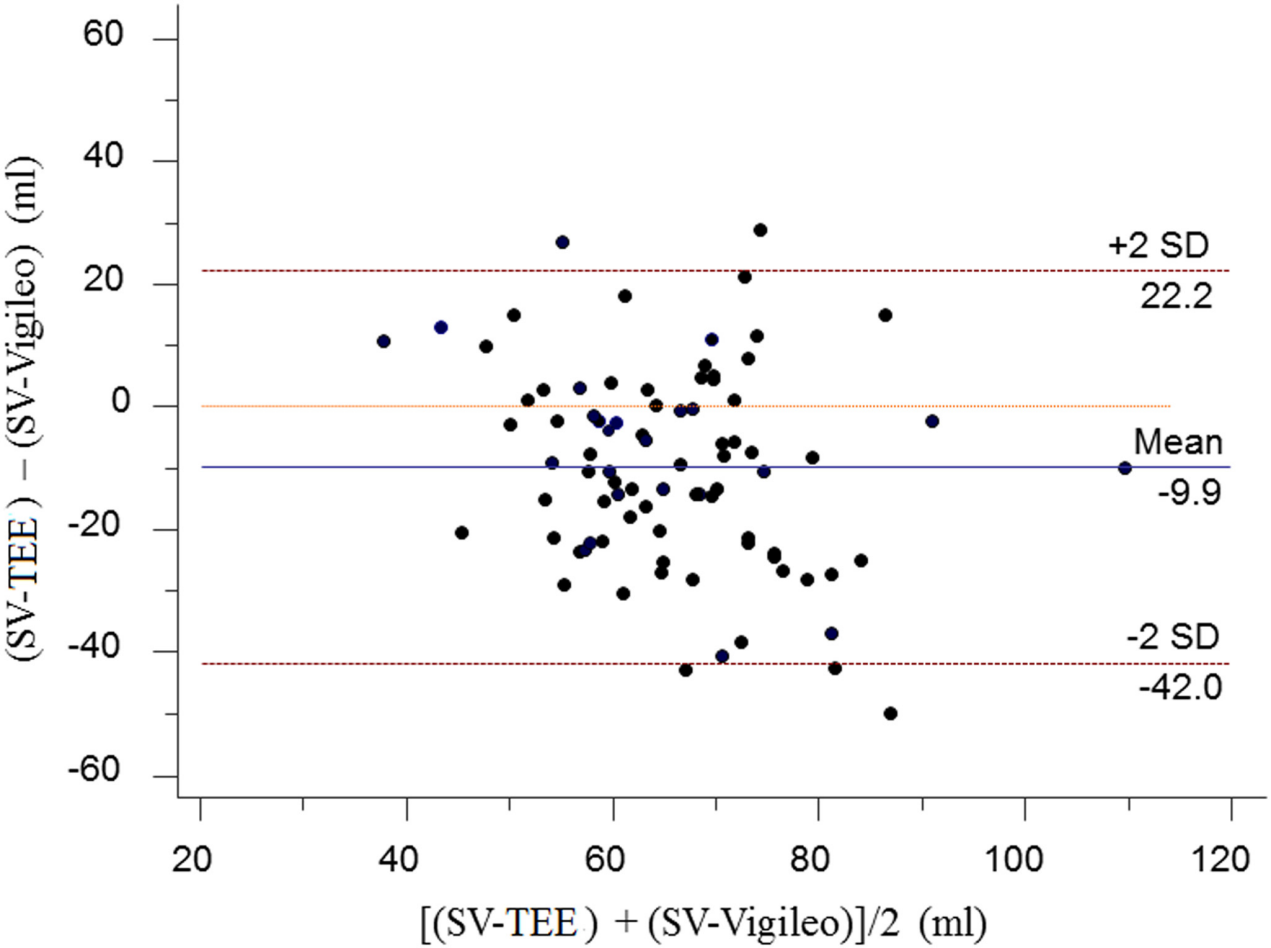
Bland-Altman plot for the difference between the SV-TEE and the SV-Vigileo during pneumoperitoneum combined with the steep Trendelenburg position. SV-TEE, stroke volume measured using transesophageal echocardiography; SV-Vigileo, stroke volume derived by the third-generation FloTrac/Vigileo^™^ system.

During pneumoperitoneum combined with the steep Trendelenburg position, 84 paired data were collected (42 from T_1_ and 42 from T_2_). Bland-Altman analysis for repeated measures showed a mean bias of -9.9 ml, a 95% limit of agreement of -42.0 to 22.2 ml, and a percentage error of 53.8% (Fig 1), implying that the ability of the third-generation FloTrac/Vigileo^™^ system to measure SV was not acceptable during pneumoperitoneum combined with the Trendelenburg position. In addition, the percentage error was 44.9%, 57.2%, and 50.3% at T_0_, T_1_, and T_2_, respectively (Table 3).

**Table 3:** Bland-Altman analyses of the SV-Vigileo compared with the SV-TEE.

	T_0_	T_1_	T_2_
Bias			
Mean (ml)	-5.4	-11.7	-8.1
SD (ml)	13.7	16.3	16.3
95% LOA (ml)	-32.3 to 21.5	-43.6 to 20.2	-40.0 to 23.9
Lower LOA (ml)	-39.7 to -24.9	-52.4 to -34.8	-48.8 to -31.2
Upper LOA (ml)	14.1 to 28.9	11.4 to 29.0	15.1 to 32.7
Percentage error (%)	44.9	57.2	50.3

T_0_, after anesthetic induction in supine position; T_1_, 3 minutes after the steep Trendelenburg position (35°) was added to pneumoperitoneum during which time insufflation pressure was set to 15 mmHg; T_2_, 3 minutes after 500 ml of colloid infusion in T_1_; SV-Vigileo, stroke volume derived by the FloTrac/Vigileo^™^ system; SV-TEE, stroke volume measured using transesophageal echocardiography; LOA, limit of agreement.

**Fig 2:**
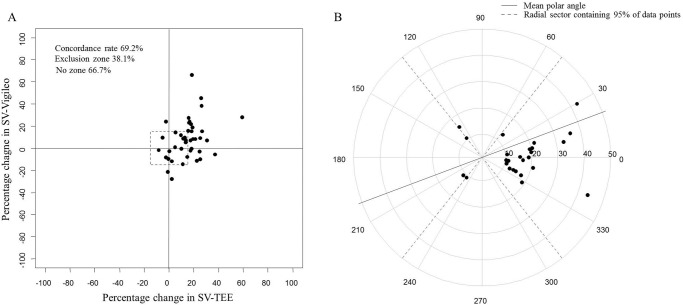
The four-quadrant plot and polar plot analysis. These figures identify an unreliable ability of the third-generation FloTrac/Vigileo^™^ system to track changes in the stroke volume after fluid administration in the high systemic vascular resistance state observed during laparoscopic prostatectomy. (A) The four-quadrant plot analysis shows a concordance rate of 69.2%, when applying an exclusion zone of 15%. (B) The polar plot analysis shows a mean polar angle of 20.6° and a radial sector containing 95% of the data points of ±51.5%, when applying an exclusion zone of 10%.

In the four-quadrant plot and polar plot analysis (Fig 2), the trending ability between T_1_ and T_2_ of the third-generation FloTrac/Vigileo^™^ system to track changes in SV after fluid administration was not good during pneumoperitoneum combined with the steep Trendelenburg position. The four-quadrant plot analysis showed a concordance rate of 69.2% (95% confidence interval 51.5–87.0%). The polar plot analysis showed a mean polar angle of 20.6°, a SD of the polar angle of 16.4°, and a radial sector containing 95% of the data points of ± 51.5°. The angular concordance rate in polar plot analysis was 76.9% (95% confidence interval 60.7–93.1%).

**Fig 3:**
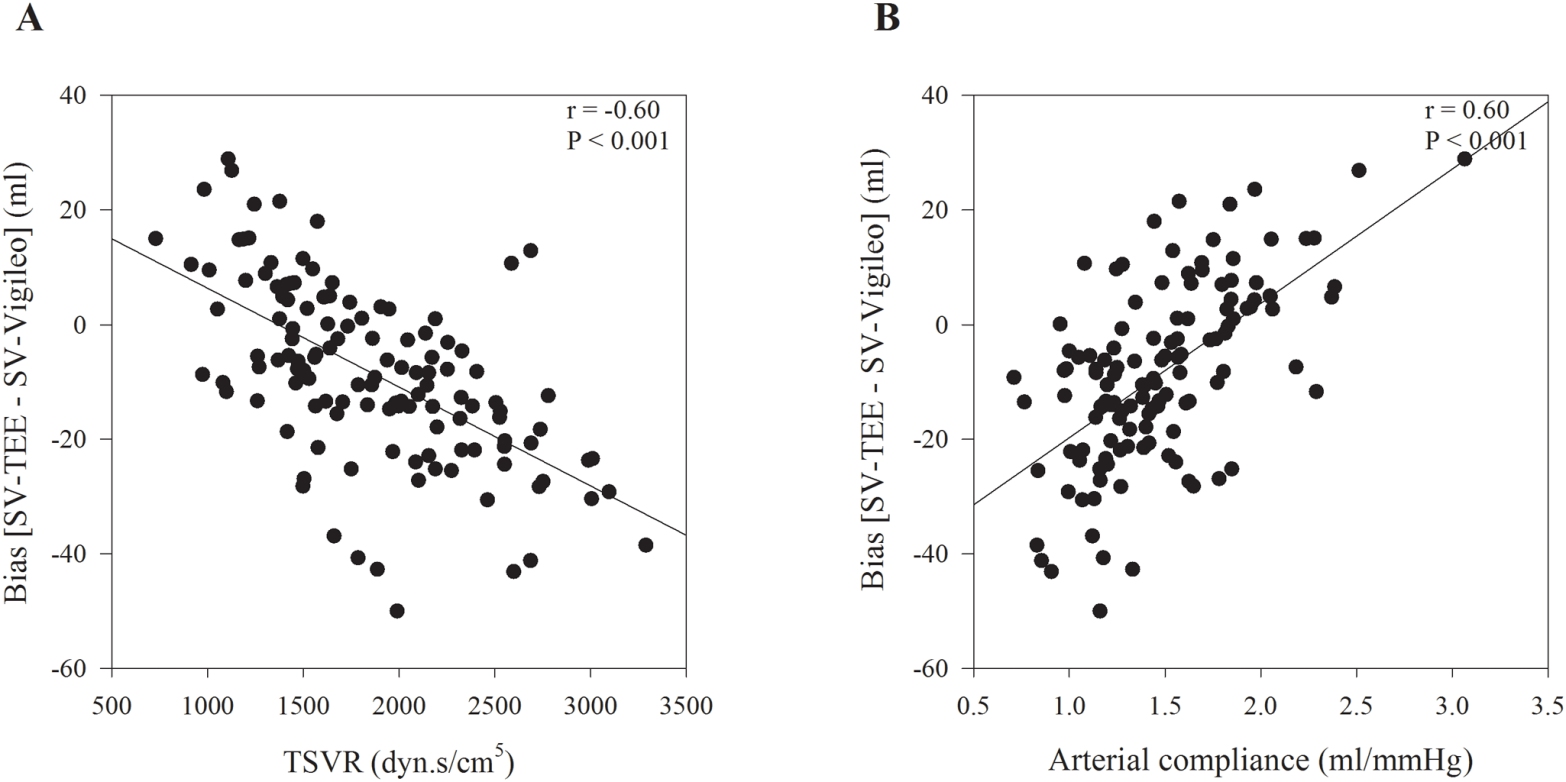
Correlation analyses (A) between total systemic vascular resistance (TSVR) and the differences between the stroke volumes measured using transesophageal echocardiography (SV-TEE) and the third-generation FloTrac/Vigileo^™^ system (SV-Vigileo) and (B) between arterial compliance the differences between the SV-TEE and the SV-Vigileo.

There was a significant correlation between arterial load (TSVR and arterial compliance) and the difference between the SV-TEE and the SV-Vigileo (Fig 3). The difference between the SV-TEE and the SV-Vigileo decreased with increasing TSVR (CC = -0.60, P < 0.001), and decreased with decreasing arterial compliance (CC = 0.60, P < 0.001).

## Discussion

In our present study, we found that the third-generation FloTrac/Vigileo^™^ system was not reliable in measuring the SV or in tracking changes in the SV after fluid administration in the high SVR state observed in patients undergoing laparoscopic prostatectomy. In addition, we found that the more the SVR increased, the more the third-generation FloTrac/Vigileo^™^ system overestimated the SV. Our results suggest that the use of the third- generation FloTrac/Vigileo^™^ system might be limited for optimizing hemodynamics in patients with a high SVR during laparoscopic surgery.

Several monitoring systems have been introduced for cardiac output measurement using pulse contour analysis, including the PiCCO^™^ system, LiDCO^™^ system, and FloTrac/Vigileo^™^ system [12,13]. An external calibration using transpulmonary dilution techniques is required in the PiCCO^™^ system and the LiDCO^™^ system (LiDCO^™^ plus) [12]. It has been reported that these systems showed acceptable performance for cardiac output measurement using pulse contour analysis [14,15]. In contrast, the FloTrac/Vigileo^™^ system internally calibrates the factor reflecting the arterial tone using the patient’s demographic data and the shape of the arterial waveform to calculate the SV, without an external calibration [2]. An inadequate performance of this system has been shown in patients with a low SVR [16,17]. To resolve this drawback, a large data set that included patients with liver cirrhosis or sepsis was collected to develop the third-generation FloTrac/Vigileo^™^ system, which, nonetheless, still showed an unacceptable performance in measuring the SV and in tracking changes in the SV [3–6]. Similarly, it has been recently reported that the third-generation FloTrac/Vigileo^™^ system was not reliable in measuring the SV and in tracking SV changes after phenylephrine administration in cardiac surgical patients, irrespective of the SVR states [18,19]. To adjust an acute change in arterial load, the latest fourth-generation FloTrac/Vigileo^™^ system was introduced, in which the calibration factor is more updated than in the third-generation system [20,21]. Few studies have reported that the fourth-generation FloTrac/Vigileo^™^ system showed an improved ability to track changes in the SV after phenylephrine administration, though not reaching good tracking ability [20,21]. It is notable that the tracking abilities of SV changes in pulse contour analysis after fluid or vasopressor administration have been reported to be different, with a better performance seen after fluid administration than after vasopressor administration, because of a less change in arterial load after fluid administration [8]. Furthermore, elucidation of the ability of the third-generation FloTrac/Vigileo^™^ system to track SV changes after fluid administration is required for it to be used in goal-directed therapy, which improves perioperative outcomes [22]. Our present study is the first to indicate that the third-generation FloTrac/Vigileo^™^ system does not reliably track changes in SV after fluid administration in the high SVR state during laparoscopic surgery. Pneumoperitoneum performed during laparoscopic surgery has been known to increase SVR [23]. Indeed in our current analyses, the SVR was high during pneumoperitoneum combined with the Trendelenburg position, and it still remained high although SVR decreased after fluid administration, which is probably attributable to reduced blood viscosity induced by hemodilution [24].

In addition, a significant correlation between the TSVR and the difference between the SV-TEE and the SV-Vigileo was identified in the present study. Our results showed that the SV-Vigileo was higher than the SV-TEE in a high SVR state. A previous study described a similar correlation between the SVR and the difference between the SV measured using the reference method and the SV-Vigileo in cirrhotic patients who showed a low SVR [3]. However, that previous study showed lower SV-Vigileo compared with the SV measured using the reference method [3], which contrasts to our current results. Taken together, the cumulative evidence to date suggests that the SV-Vigileo might be affected by a wide range of SVR, showing that the FloTrac/Vigileo^™^ system overestimates the SV in high SVR state, while it underestimates the SV in a low SVR state.

The arterial tone, which is calculated from the patient’s demographic data and the shape of the arterial waveform in the FloTrac/Vigileo^™^ system [2], is composed of both steady (SVR) and pulsatile (arterial compliance) components [25,26]. However, previous studies investigating the reliability of the SV measured using the FloTrac/Vigileo^™^ system have concentrated on the SVR, ignoring the arterial compliance, which is a key determinant of the relationship between the SV and arterial waveform [5]. Our current study has found that the arterial compliance as well as the TSVR correlated with the difference between the SV-TEE and the SV-Vigileo, suggesting the effect of these two aspects of arterial tone on the ability of the third-generation FloTrac/Vigileo^™^ system to measure the SV. Our findings in this regard are consistent with those of a recent report that each aspect of arterial load influences the difference between the SV measured using pulse contour analysis and the reference method [8].

Our study had the following limitations. First, transesophageal echocardiography is not the gold standard method for measuring SV, raising the possibility of variations when compared with the thermodilution technique, or the gold standard method. However, the cardiac output measured by transesophageal echocardiography has been reported to be in very good agreement with the cardiac output derived using the thermodilution technique [27,28]. In addition, it has been known that the coefficient of variation of aortic Doppler echocardiography for SV measurement was 7.8% [29]. The precision of aortic Doppler echocardiography for SV measurement, calculated as twice the coefficient of variation, is 15.6% [30], similar to the 20% for the thermodilution technique. Moreover, the insertion of a pulmonary artery catheter, which has not been determined to be beneficial even in high-risk surgical patients [31], might raise an ethical issue in our patients. Second, the post hoc nature of our analysis was a potential limitation. The number of data sets in our study was not determined a priori. A limited number of data sets were used in our analysis for evaluating the tracking ability. However, the upper limit of the 95% confidence interval for the concordance rate in four-quadrant plot analysis and for the angular concordance rate in polar plot did not reach the threshold for good tracking. Thus, the clinical relevance of these findings might not have been attenuated. Third, our measurements during pneumoperitoneum combined with the Trendelenburg position were obtained at one point. It remains to be determined whether the unreliable performance of the third-generation FloTrac/Vigileo^™^ system improves over time during pneumoperitoneum combined with the Trendelenburg position.

In conclusion, in a high SVR state, the third-generation FloTrac/Vigileo^™^ system overestimates the SV, and importantly, does not reliably track changes in the SV after fluid administration. These results suggest that the third-generation FloTrac/Vigileo^™^ system may not be useful for optimizing hemodynamic variables in patients with a high SVR, who need advanced cardiovascular monitoring during laparoscopic surgery.

## Supporting Information

S1 Dataset This file is the dataset of our study. (XLSX)
